# Patient-reported tolerability of endoscopic retrograde cholangiopancreatography using conscious sedation in patients with underlying depression and anxiety

**DOI:** 10.1186/s41687-026-01094-1

**Published:** 2026-06-08

**Authors:** Alejandra Tepox-Padrón, Renée El-Gabalawy, Shane Cartwright, Sangmin Lee, Priyanka Gill, Pooja Mishra, Jessica Hammal, Angelica Rivas, Jonathan Besney, Sunil Samnani, Howard Guo, Getanshu Malik, Mehul Gupta, Catherine Tsai, Arjun Kundra, Robert J. Hilsden, Nauzer Forbes

**Affiliations:** 1 Division of Gastroenterology and Hepatology, University of Calgary, Calgary, Canada; 2 Department of Community Health Sciences, University of Calgary, Calgary, Canada; 3 Department of Clinical Health Psychology, University of Manitoba, Winnipeg, Canada; 4 Department of Anesthesiology, Perioperative and Pain Medicine, University of Manitoba, Winnipeg, Canada; 5 Cumming School of Medicine, University of Calgary, Calgary, Canada; 6 Department of Medicine, University of Calgary, Calgary, Canada; 7 Department of Gastroenterology and Hepatology, University of Virginia, School of Medicine, Charlottesville, USA

**Keywords:** Endoscopic retrograde cholangiopancreatography, Mental health, Patient-reported experience measures, Perioperative mental health.

## Abstract

**Background:**

Endoscopic retrograde cholangiopancreatography (ERCP) is often performed under conscious sedation, which may increase pain and dissatisfaction. Mental health conditions may influence patient-reported experience measures (PREMs), including tolerability. This study explores the association between pre-existing anxiety and/or depression and ERCP tolerability using the validated Patient-Reported Scale for Tolerability of Endoscopic Procedure (PRO-STEP) to address this.

**Methodology:**

We performed a retrospective analysis of prospectively maintained data from an international observational cohort of adult patients undergoing ERCP. Pre-existing anxiety/depression were identified prior to the index procedure and the PRO-STEP questionnaire was used to evaluate peri- and post-procedure outcomes. Univariable and multivariable logistic regressions examined the associations between pre-operative anxiety/depression and peri- and post-operative tolerability and patient-reported health outcomes.

**Results:**

Among 3,714 participants, 13% had anxiety and/or depression. The mean age of participants in the control group was 62.3 ± 17.4 years, and 49.9% were female, while in the group with depression and/or anxiety, the mean age was 60.0 ± 16.4 years and 68.3% were female (*p* < 0.001). Common bile duct stones were the most common indications for ERCP in both groups (41.6% of controls and 42.4% of the depression/anxiety group, *p* = 0.10). Patients in the depression/anxiety group reported higher rates of opioid use (23.4% vs. 13.8%, *p* < 0.001), cannabis use (22.8% vs. 10.5%, *p* < 0.001), and heavy alcohol consumption (5.0% vs. 3.6%, *p* < 0.001). There were no statistically significant differences between groups in terms of disposition, comorbidities, or procedural parameters. Underlying anxiety and/or depression was significantly associated with increased intra-procedural awareness score > 3 (odds ratio, OR, 1.55, 95% CI 1.23–1.95) and discomfort score > 6 (OR 1.73, 95% CI 1.23–2.43) and with post-procedural scores > 3 for abdominal pain (OR 1.44, 95% CI 1.08–1.93), nausea (OR 2.03, 95% CI 1.43–2.89), and distension (OR 2.12, 95% CI 1.29–3.50).

**Conclusions:**

Patients with pre-existing anxiety and/or depression reported significantly worse tolerability of ERCP under conscious sedation. Although further research is needed in this area, staff in gastrointestinal endoscopy units should consider strategies aimed at improving tolerability and, consequently, satisfaction among vulnerable populations.

**Supplementary Information:**

The online version contains supplementary material available at 10.1186/s41687-026-01094-1.

## Background

Depression and anxiety are the most prevalent mental health conditions globally and can lead to severe functional impairment [1, 2]. Patients with depression and/or anxiety experience worse post-operative outcomes across various surgical settings [3], including reduced functional recovery and higher rates of peri- and post-operative pain [4]. In gastrointestinal endoscopy, feelings of anxiety in the peri-procedural period have been associated with increased sedation requirements, longer sedation time, and delayed post-procedural recovery [5], which collectively impact both patient and endoscopist satisfaction [6]. The presence of a pre-existing diagnosis of depression and/or anxiety could therefore predispose to such feelings.

Endoscopic retrograde cholangiopancreatography (ERCP) is a widely performed procedure in the management of several pancreaticobiliary conditions [7]. These include biliary stones, pancreatic, biliary, or gallbladder cancers, or pancreatitis. ERCP involves passage of a long tube with a camera at the end of it through the mouth into the first part of the small bowel, where the opening(s) to the bile duct and/or pancreatic duct can be found. A high rate of adverse events (AEs) has consistently been associated with ERCP [8], which is variably performed under conscious sedation, deep sedation, or general endotracheal anaesthesia. Though all approaches have been shown to be safe and effective, variations in local resource availability results in conscious sedation being a widely used approach to support ERCP in many healthcare regions around the world [9].

Though up to one third of patients undergoing ERCP under conscious sedation have been shown to develop abdominal pain or discomfort in the days following the procedure [10], decidedly less is known regarding intra- and immediate post-procedural tolerability of the procedure and risk factors for these outcomes. Various mechanisms could explain potentially poorer tolerability of ERCP among patients with pre-existing depression and/or anxiety. Patients experiencing pre- and/or peri-procedural ‘state’ anxiety at the time of endoscopic procedures present a challenge in terms of sedation [11]. Individuals with baseline depression and/or anxiety, who are in turn at risk of higher peri-procedural state anxiety, may exhibit reduced responsiveness to sedatives, potentially leading to increased awareness during ERCP. Moreover, pain and depression are processed through similar neurological pathways, and this overlap can result in exacerbated symptoms and reduced responsiveness to pain treatments [12]. Thus, pre-existing depression and/or anxiety in patients undergoing ERCP may decrease the effectiveness of analgesics administered during the procedure with conscious sedation, and increase sensations of discomfort, as well as somatic and visceral pain, including abdominal pain and distention. Finally, high levels of anxiety are positively correlated with increased awareness of somatic symptoms, such as pain and nausea, as well as greater subjective complaints [13]. Therefore, patients with depression/anxiety, particularly those with anxiety, might experience more nausea around the time of the procedure.

Patient-reported experience measures (PREMs) [14] are tools used to assess and report patients’ perceptions of their own healthcare encounters. Using PREMs can improve healthcare quality and performance [15], enhance our understanding of patient values and preferences, facilitate informed clinical decision-making, and foster improved communication [16]. In patients with depression and/or anxiety, enhanced inpatient satisfaction has shown to correlate with improved symptoms [17]. However, there remains a knowledge gap regarding the potential associations between pre-existing depression/anxiety and tolerability of ERCP. Therefore, we aimed to examine the association between pre-existing depression and/or anxiety and patient-reported procedural tolerability of ERCP performed under conscious sedation.

## Methods

### Setting and participants

We conducted an analysis of prospective multi-centre data on patients undergoing ERCP for any indication [18] across nine centres in Canada, the United States, and Europe. The study aimed to include patients from two groups: (1) those with pre-existing depression and/or anxiety and (2) those without pre-existing diagnoses of either depression or anxiety. Data were collected in real time intra-procedurally and recorded by trained research assistants at all study sites. Following their procedure, patients were assessed by research assistants to describe their intra- and immediately post-procedural experiences according to a validated endoscopy-specific PREM (see below). All participants provided informed consent. The study was approved by the Conjoint Health Research Ethics Board (CHREB) at the University of Calgary (REB23-0942) and at each participating site.

### Exposures and covariates

The primary exposure variable was a pre-existing diagnosis of one or both of depression and/or anxiety. A patient was classified as having the exposure if (1) a formal history of either diagnosis was documented on their medical record or (2) if any psychiatric medication use was noted in the chart, in which case the patient was asked via interview what the indication was in order to confirm a diagnosis. No further eligibility criteria were applied regarding length and/or severity of diagnoses, active treatment, or any other disease-specific parameters.

Covariates were selected based on their plausible associations with the outcomes or exposures, including: age, sex, patient disposition, smoking status, baseline consumption/use of alcohol, opioids, and cannabinoids [19], comorbidity status, indications for ERCP, previous ERCP status, sphincterotomy, size of the common bile duct, pancreatic duct cannulation, pancreatogram, biliary stricture, stent deployment, and difficult cannulation (5 or more cannulation attempts, five or more minutes or two or more unintentional pancreatic wire passages) [20].

### Outcomes

The primary outcome was patient-reported tolerability of ERCP under conscious sedation, as measured and reported using the Patient-Reported Scale for Tolerability of Endoscopic Procedures (PRO-STEP) [21]. This PREM was designed to be administered immediately prior to discharge following endoscopic procedures in patients receiving endoscopist-directed conscious sedation. It comprises two domains: the first assesses intra-procedural tolerability (with specific questions on awareness and discomfort), while the second evaluates post-procedural tolerability at discharge (with specific questions on nausea, distention, abdominal pain, and throat pain). Each question is rated on a Likert scale from 0 to 10. For the purposes of this study, we performed comparisons of mild versus moderate/severe scores and comparisons of mild/moderate versus severe scores [21, 22].

### Statistical analysis

Descriptive statistics, specifically, frequencies and percentages for categorical variables and means with standard deviations or medians and interquartile ranges for continuous variables, were used to present baseline characteristics. Student’s T-tests or Wilcoxon rank-sum tests were used for continuous variables, and chi-square tests, Fisher’s exact tests, or Kruskal-Wallis tests were used for categorical variables.

Logistic regression modelling was employed to elucidate potential associations between pre-existing depression and/or anxiety and each of the categorical outcomes of patient-reported tolerability of ERCP. Multivariable logistic regression modelling was performed to explore associations between the primary exposure and each PRO-STEP sub-score along with potentially relevant covariates. Covariates retained for the final models were informed by both statistical and clinical significance and were selected using backward elimination with a significance level of < 0.20. Sensitivity analyses were performed to evaluate associations between anxiety only and depression only and relevant outcomes, and to examine associations between anxiety/depression and outcomes in outpatients only. Results were expressed using adjusted odds ratios (aORs). The statistical significance threshold was set at 0.05, with 95% confidence intervals (CIs) reported alongside ORs. All analyses were performed using Stata v18 (StataCorp, College Station, TX).

## Results

### Overview and descriptive statistics

A total of 3,714 participants underwent ERCP with conscious sedation between September 2018 and September 2023 and were assessed in the post-procedure setting using PRO-STEP. Among these, 3,231 (87.0%) were classified as controls, while 483 (13.0%) had underlying depression and/or anxiety. The mean age of the participants in the control group was 62.3 ± 17.4 years, and 49.9% were female, while in the group with depression and/or anxiety, the mean age was 60.0 ± 16.4 years and 68.3% were female (*p* < 0.001). Suspected common bile duct stones were the most common indications for ERCP in both groups (41.6% of controls and 42.4% of the depression/anxiety group, *p* = 0.10). Patients in the depression/anxiety group reported higher rates of opioid use (23.4% vs. 13.8%, *p* < 0.001), cannabis use (22.8% vs. 10.5%, *p* < 0.001), and heavy alcohol consumption (5.0% vs. 3.6%, *p* < 0.001) compared to the control group. There were no statistically significant differences between groups in terms of disposition, comorbidities, or procedural parameters. Table 1 summarizes these results.

**Table 1:** Baseline demographic, clinical and procedural characteristics of ERCP

	Total 3,714 (100.0%)	Control group 3,231 (87.0%)	Depression and/or anxiety 483 (13.0%)	*p* value
**Baseline Demographic and Clinical Characteristics**				
Age (years), mean (SD)	62.0 (17.3)	62.3 (17.4)	60.0 (16.4)	**< 0.001**
Sex, n (%)				
Male	1,768 (47.7)	1,616 (50.1)	152 (31.5)	**< 0.001**
Female	1,938 (52.3)	1,608 (49.9)	330 (68.3)	
Anxiety, n (%)		0 (0.0)	284 (58.8)	
Depression, n (%)		0 (0.0)	335 (69.4)	
Disposition, n (%)				
Outpatient	2,168 (58.4)	1,899 (58.8)	269 (55.7)	0.576
Inpatient	1,545 (41.6)	1,331 (41.2)	214 (44.3)	
Intensive care	1 (< 0.1)	1 (< 0.1)	0 (0.0)	
Charlson Comorbidity Index, mean (SD)	3.2 (2.7)	3.2 (2.8)	3.2 (2.6)	0.149
Procedural indication, n (%)				
CBD stones	1,550 (41.7)	1,345 (41.6)	205 (42.4)	0.097
Stent removal, exchange	766 (20.6)	668 (20.7)	98 (20.3)	
CBD obstruction	683 (18.4)	610 (18.9)	73 (15.1)	
Pancreatic indication	66 (1.8)	50 (1.5)	16 (3.3)	
Other	649 (17.5)	558 (17.3)	91 (18.8)	
Opioid use, n (%)	557 (15.0)	444 (13.8)	113 (23.4)	**< 0.001**
Cannabis use, n (%)	450 (12.1)	340 (10.5)	110 (22.8)	**< 0.001**
Alcohol consumption, n (%)				
None	804 (21.7)	686 (21.3)	118 (24.5)	**< 0.001**
Former, noncurrent	1,072 (28.9)	911 (28.2)	161 (33.4)	
Occasional	1,213 (32.7)	1,082 (33.6)	131 (27.2)	
Moderate	479 (12.9)	431 (13.4)	48 (10.0)	
Heavy	139 (3.7)	115 (3.6)	24 (5.0)	
Procedural Characteristics of ERCP				
Previous ERCP, n (%)	1,704 (45.9)	1,478 (45.8)	226 (46.8)	0.197
Sphincterotomy, n (%)	1,810 (52.6)	1,584 (52.7)	226 (52.3)	0.488
Targeted PD, n (%)	212 (5.7)	177 (5.5)	35 (7.3)	0.317
Pancreatic cannulation, n (%)	741 (20.6)	627 (20.1)	114 (24.5)	0.553
Pancreatogram partial/full, n (%)	255 (6.9)	215 (6.7)	40 (8.3)	0.224
Stricture present, n (%)	903 (27.0)	791 (27.1)	112 (26.5)	0.563
Bile duct size, n (%)				
6 mm or less	368 (12.5)	321 (12.7)	47 (11.6)	0.374
7 to 8 mm	600 (20.4)	517 (20.5)	83 (20.4)	
9 to 10 mm	785 (26.8)	681 (26.9)	104 (25.6)	
11 to 14 mm	821 (28.0)	690 (27.3)	131 (32.3)	
15 to 19 mm	269 (9.2)	240 (9.5)	29 (7.1)	
20 mm or greater	91 (3.1)	79 (3.1)	12 (3.0)	
Metal stents, n (%)	363 (9.8)	321 (9.9)	42 (8.7)	0.348
Difficult cannulation, n (%)	1,049 (28.2)	905 (28.0)	144 (29.8)	0.705

Abbreviations: ERCP, endoscopic retrograde cholangiography; CBD, common bile duct; SD, standard deviation; PD, pancreatic duct

^a^p-value obtained after chi-square or Fisher’s exact test for categorical variables, and Student-T test for continuous variables

### Underlying depression and/or anxiety and procedural tolerability

PRO-STEP scores compared between participants with underlying depression/anxiety and control patients are presented in Table 2. In the intra-procedural domains, patients with underlying depression/anxiety had higher mean scores for awareness (2.0 ± 3.3 vs. 1.5 ± 2.8, respectively; *p* = 0.01) but similar scores for discomfort (1.4 ± 2.8 vs. 1.0 ± 2.3, respectively; *p* = 0.11) compared to controls. Additionally, in the depression/anxiety group, there was a significantly higher proportion of participants who experienced both moderate-to-severe awareness compared to controls (score > 3: 25.5% vs. 18.7%, *p* < 0.001) and severe awareness compared to controls (score > 6: 15.1% vs. 10.3%, *p* = 0.003). These statistically significant differences were also observed for moderate-to-severe discomfort (score > 3: 17.6% vs. 13.6%, *p* = 0.02) and severe discomfort (score > 6: 9.9% vs. 5.6%, *p* < 0.001). In the post-procedural domains, both groups reported mean scores of less than 1 for abdominal pain, throat pain, nausea, and distension. However, a statistically significant higher proportion of participants in the depression/anxiety group reported significant abdominal pain (score > 3: 15.5% vs. 10.2%, *p* < 0.001) and distension (score > 3: 5.5% vs. 3.3%, *p* = 0.03) at discharge, compared to controls. Proportions of patients experiencing significant nausea were also significantly higher among patients with underlying depression and/or anxiety (mean score: 0.7 ± 2.0 vs. 0.4 ± 1.4, *p* = 0.04, score > 3: 9.9% vs. 4.3%, *p* < 0.001, and score > 6: 4.4% vs. 2.1%, *p* = 0.006).

**Table 2:** PRO-STEP scores compared between control patients and those with depression and/or anxiety

	Control group 3,231 (87%)	Depression and/or anxiety 483 (13%)	*p* value^a^
Intra-procedural domains			
*Self-reported awareness*,* mean (SD)*	1.5 (2.8)	2.0 (3.3)	**0.013**
Score > 3, n (%)^b^	598 (18.7)	122 (25.5)	**< 0.001**
Score > 6, n (%)^b^	331 (10.3)	72 (15.1)	**0.003**
*Discomfort Level*,* mean (SD)*	1.0 (2.3)	1.4 (2.8)	0.111
Score > 3, n (%)^b^	434 (13.6)	84 (17.6)	**0.024**
Score > 6, n (%)^b^	178 (5.6)	48 (9.9)	**< 0.001**
Post-procedural domains			
*Abdominal Pain*,* mean (SD)*	0.8 (2.0)	1.1 (2.4)	0.065
Score > 3, n (%)^b^	329 (10.2)	74 (15.5)	**< 0.001**
Score > 6, n (%)^b^	142 (4.4)	30 (6.3)	0.079
*Throat Pain*,* mean (SD)*	0.5 (1.3)	0.5 (1.5)	0.528
Score > 3, n (%)^b^	149 (4.6)	25 (5.2)	0.563
Score > 6, n (%)^b^	37 (1.2)	8 (1.7)	0.367
*Nausea Level*,* mean (SD)*	0.4 (1.4)	0.7 (2.0)	**0.040**
Score > 3, n (%)^b^	139 (4.3)	48 (9.9)	**< 0.001**
Score > 6, n (%)^b^	68 (2.1)	21 (4.4)	**0.006**
*Distention Level*,* mean (SD)*	0.3 (1.1)	0.4 (1.4)	0.201
Score > 3, n (%)^b^	107 (3.3)	26 (5.5)	**0.025**
Score > 6, n (%)^b^	26 (0.8)	7 (1.5)	0.185

Abbreviations: PRO-STEP, Patient-Reported Scale for Tolerability of Endoscopic Procedures: SD, standard deviation

^a^p-value obtained after chi-square, Fisher’s exact test or Kruskal-Wallis test for categorical variables, and Wilcoxon rank-sum test for continuous variables

^b^Binary variable (e.g., yes or no) in which the reference group corresponds to the absence of that characteristic

Table 3 presents the results of univariable and multivariable logistic regression analyses for moderate-to-severe (> 3) and severe (> 6) categories of PRO-STEP as categorical outcomes. After adjustment, individuals with underlying depression and/or anxiety had increased odds of experiencing intra-procedural awareness categorized as moderate-to-severe (aOR 1.55, 95% CI 1.23–1.95; *p* < 0.001) or severe (aOR 1.40, 95% CI 1.02–1.92; *p* = 0.04) compared to controls. Pre-existing depression/anxiety was also associated with severe intra-procedural discomfort (aOR 1.73, 95% CI 1.23–2.43; *p* = 0.002) during ERCP under conscious sedation. Additionally, patients with pre-existing depression and/or anxiety experienced increased post-procedural abdominal pain (score > 3: aOR 1.44, 95% CI 1.08–1.93; *p* = 0.01) and nausea (score > 3: aOR 2.03, 95% CI 1.43–2.89; *p* < 0.001) at discharge, in addition to higher post-procedural distention (score > 3: aOR 2.12, 95% CI 1.29–3.50; *p* = 0.003, and score > 6: aOR 2.91, 95% CI 1.15–7.40; *p* = 0.02). No associations between depression/anxiety and throat pain at discharge were found.

**Table 3:** Unadjusted and adjusted odds ratios between underlying depression/anxiety and PRO-STEP scores of > 3 and > 6

Variables	PRO-STEP >3^c^			PRO-STEP >6^c^		
	Unadjusted OR (95% CI)	Adjusted OR^a^ (95% CI)	*p* value^b^	Unadjusted OR(95% CI)	Adjusted OR^a^ (95% CI)	*p* value^b^
**Intra-procedural domains**						
Awareness	1.49 (1.19, 1.87)	1.55 (1.23, 1.95)	**0.001**	1.54 (1.17, 2.03)	1.40 (1.02, 1.92)	**0.035**
Discomfort Level	1.35 (1.05, 1.75)	0.79 (0.40, 1.57)	0.508	1.89 (1.35, 2.64)	1.73 (1.23, 2.43)	**0.002**
**Post-procedural domains**						
Abdominal Pain	1.62 (1.23, 2.13)	1.44 (1.08, 1.93)	**0.014**	1.46 (0.97, 2.19)	1.18 (0.78, 1.79)	0.436
Throat Pain	1.14 (0.74, 1.75)	0.97 (0.62, 1.50)	0.889	1.46 (0.68, 3.16)	1.72 (0.75, 3.92)	0.198
Nausea	2.46 (1.75, 3.47)	2.03 (1.43, 2.89)	**0.001**	2.12 (1.29, 3.49)	1.61 (0.96, 2.68)	0.070
Distention Level	1.68 (1.08, 2.60)	2.12 (1.29, 3.50)	**0.003**	1.89 (0.79, 4.24)	2.91 (1.15, 7.40)	**0.024**

Abbreviations: ERCP, endoscopic retrograde cholangiopancreatography; PRO-STEP, Patient-Reported Scale for Tolerability of Endoscopic Procedures; OR, odds ratio; CI, confidence intervals

^a^Adjusted for Age and sex, and potentially one or more of these covariates: disposition, Charlson comorbidity index, indication, opioids, cannabis, alcohol consumption, previous ERCP, sphincterotomy, targeted duct, size of the CBD, pancreatic cannulation, pancreatogram, stricture present on ERCP, metal stent deployed, difficult cannulation

^b^p-value associated with adjusted OR in logistic regression model

^c^Binary variable (e.g., yes or no) in which the reference group corresponds to the absence of that characteristic

In the sensitivity analysis of patients with depression alone, patients experienced higher levels of intra-procedural awareness (score > 6: aOR 1.62, 95% CI 1.18–2.22; *p* = 0.003) and discomfort (score > 6: aOR 1.60, 95% CI 1.06–2.39; *p* = 0.02) compared to the cohort without any depression or anxiety. Pre-existing depression was also associated with scores of > 3 for post-procedural abdominal pain (aOR 1.44, 95% CI 1.01–2.05; *p* = 0.04), nausea (aOR 1.97, 95% CI 1.32–2.95; *p* < 0.001), and distension (aOR 1.90, 95% CI 1.06–3.38; *p* = 0.03) following an ERCP (Supplementary Table 1). Patients with anxiety alone had increased odds of experiencing moderate-to-severe (aOR 2.00, 95% CI 1.50–2.68; *p* < 0.001) and severe intra-procedural awareness (aOR 1.76; 95% CI 1.23–2.51; *p* = 0.002) and discomfort (aOR 1.96, 95% CI 1.31–2.94; *p* = 0.002). Patients with anxiety alone also experienced moderate-to-severe post-procedural abdominal pain (aOR 1.59; 95% CI 1.06–2.38; *p* = 0.024), along with moderate-to-severe (aOR 2.19, 95% CI 1.38–3.47; *p* = 0.001) and severe nausea (aOR 3.37; 95% CI 1.73–6.56; *p* = 0.001) compared to the cohort without any depression or anxiety (Supplementary Table 2). Finally, in the subgroup of outpatients alone, individuals with depression and/or anxiety had greater odds of reporting intra-procedural nausea scores > 3 (aOR 2.20, 95% CI 1.43–3.40; *p* < 0.001), with no other statistically significant associations compared to controls (Supplementary Table 3).

## Discussion

In this cohort study of over 3,500 patients across nine centres, pre-existing diagnoses of depression and/or anxiety were significantly associated with poorer self-reported tolerability of ERCP after adjustment for relevant confounders including pre-existing substance use or misuse. Specifically, pre-existing depression and/or anxiety was associated with higher levels of intra-procedural awareness and discomfort as well as higher levels of post-procedural abdominal pain, nausea, and distention at the time of discharge from the ERCP unit.

Our findings align with those previously reported by Jeurnink et al. who reported that up to half of patients undergoing ERCP under conscious sedation experienced peri-procedural and/or post-procedural pain or discomfort, while up to 10% reported significant mental issues such as state anxiety around the time of and after the procedure [10]. However, pre-existing depression and/or anxiety have not been investigated as a potential risk factor for these self-reported outcomes in this or any other study, to our knowledge, either in the context of ERCP or more broadly in the realm of all endoscopic procedures. While state anxiety is undoubtedly an important factor in patients’ peri-procedural experience, knowledge of pre-existing depression and/or anxiety is arguably even more actionable given that these patients could theoretically be identified via pre-screening and supports or other interventions could be provided earlier on.

PREMs and patient-reported outcome measures (PROMs) are valuable tools completed by patients to evaluate their experiences and outcomes following health services [14, 23–25] but to date have been arguably underutilized in advanced endoscopy. As an example of a successfully applied PROM, the recently developed PAN-PROMISE patient-reported scale was originally developed for acute pancreatitis not related to ERCP, and was found to correlate with the resolution of acute pancreatitis, local AEs, and the development of severe pancreatitis [26]. A prospective cohort study evaluating the usefulness of PAN-PROMISE as a predictor for post-ERCP morbidity showed that an elevated score, even in the absence of post-ERCP pancreatitis, strongly correlated with lower physical quality of life scores as well as increased direct and indirect health care costs at seven days after ERCP [27]. Similarly, in our study, we employed PRO-STEP, a previously validated PREM [21] whose scores have been shown to correlate with post-ERCP outcomes [22]. More broadly, our findings validate the importance of and argue for the more widespread use of PREMs in procedural medical specialties. Routine measurement of patients’ subjective experiences and satisfaction scores has been shown to increase patients’ willingness to attend repeat procedures [28]. As a consequence, PREMs have also been shown to reduce misuse of health care services as well as medicolegal claims [28, 29].

Importantly, though the vast majority (> 90%) of control patients reported next to no abdominal pain, distention, nausea, or sore throat following their procedure (and were therefore considered ‘symptom-free’ at the time of discharge), this proportion was significantly lower for patients with pre-existing depression and/or anxiety. Given that higher abdominal pain scores have been associated with increased unplanned hospital encounters, even in the absence of post-ERCP AEs [22], it is worth exploring whether a higher occurrence of abdominal pain, nausea, and/or distension even after discharge among participants with depression and/or anxiety could partially explain this unplanned healthcare usage. If so, targeted interventions could be instituted for patients with underlying depression/anxiety undergoing ERCP, such as pre-procedural psychoeducation or planned phone calls and/or visits on the day(s) following ERCP to assess symptoms and provide reassurance or further action where needed to try to reduce the burden of unplanned healthcare usage [30]. One can also reasonably ask the question of whether our findings should prompt changes in unit-based policy whereby patients with underlying diagnoses of depression and/or anxiety should only undergo ERCP with either deep sedation or under general anesthesia.

As discussed earlier, various factors explain our findings of poorer tolerability of ERCP among patients with pre-existing depression and/or anxiety, including higher rates of pre- and peri-procedural state anxiety and lower responsiveness to conscious sedation. In addition to these, cognitive biases such as catastrophizing, maladaptive appraisals, and intolerance of uncertainty are common in patients with underlying depression and/or anxiety, and may influence and exacerbate rumination about fearful and threatening stimuli during ERCP procedures [31]. Heightened awareness during surgical procedures can intensify negative emotions, including feelings of helplessness, panic, vulnerability, fear of pain, actual physical pain, feelings of abandonment and betrayal from healthcare providers. This constellation is important given that it can also increase susceptibility to adverse psychological outcomes including post-traumatic stress disorder (PTSD) and dissatisfaction, which may have critical negative downstream consequences on future healthcare encounters [32]. Patients experiencing adverse events during or following an ERCP report more frequent dissatisfaction regarding the attitude and technical expertise of the endoscopists and are less likely to return to the same physician and hospital for a future procedure [33]. Thus, further research is needed to assess whether the reduced tolerability reported by patients with underlying depression/anxiety during ERCP has a broader negative impact on patient satisfaction, willingness to undergo repeat procedures, future sedation levels and/or plans, use of anaesthesia-guided sedation, loss of work and/or school time, and/or direct and indirect healthcare costs. To accomplish these goals, there is a need for future studies that employ validated PREMs and PROMs not only at discharge but also in the days following the procedure. ERCP is an ideal scaffold for this type of inquiry given its established relatively high adverse event rates [8].

Our study has limitations that need to be considered. Firstly, despite the large number of patients undergoing ERCP that were included, the number of participants with depression/anxiety undergoing ERCP under conscious sedation was ultimately less than 500. As a result, the proportion of patients reporting scores greater than 3 and 6 was low overall, which may explain the lack of significance in most outcomes in the sensitivity analysis of ambulatory patients. Although our study may have been underpowered to detect true associations, the low degree of variability in most of our primary estimates when considering the narrow 95% confidence intervals is encouraging that the signals we observed represent the truth. Secondly, despite using highly granular data including hundreds of potential covariates, we were unable to examine the tolerability of ERCP among patients with state anxiety or psychological distress (i.e.: a temporary phenomenon only around the time of the procedure rather than a pre-existing diagnosis of anxiety). Similarly, we did not evaluate the potentially ‘dose-dependent’ effect of the severity of depression and anxiety symptoms, nor did we account for disease chronicity and activity, and medications used, among other important factors. Thirdly, patients without formal diagnoses of depression and/or anxiety in their medical records could have been misclassified as controls if they had undiagnosed depression and/or anxiety. Fourthly, our study focused on depression and anxiety, and therefore, it remains unclear whether these associations are also potentially applicable to other mental health conditions. Additionally, our methodology exposed our results to potential biases, despite our best efforts. For instance, given that research assistants were aware of patients’ mental health histories, confirmation bias could have played a role in our findings. Although all data collection was meant to be objective and protocolized, this could have been a factor. Furthermore, additional biases may have been present in the measurement of the patient-reported outcomes. Given that asking people about negative feelings and peri- and post-procedural side effects could theoretically increase awareness and hypervigilance effects in and of itself [34], the process of requesting patient-reported experiences itself could influence the reported results, especially given that patients undergoing conscious sedation may not remember things clearly, creating a sort of recall bias. Social desirability bias could also play a role in our findings, given that patient-reported experiences were requested verbally by research assistants rather than via anonymized written or web-based forms [35]. Though we made the decision to actively solicit these results verbally to enhance participation and minimize attrition, we acknowledge that this approach could also introduce bias. Finally, we did not evaluate other crucial outcomes that could have arisen because of poorer procedural tolerability, including exacerbation of PTSD, missed time away from work or reduced quality of life.

Our study also has several strengths. To our knowledge, ours is the only study to date that specifically aimed to evaluate multiple intra- and post-procedural patient-reported domains among participants with pre-existing depression and/or anxiety undergoing ERCP. We utilized a PREM specifically validated for use in endoscopy (and shown to correlate with ERCP outcomes) to evaluate the patient’s subjective experience of ERCP under conscious sedation. We consider our data internally valid and all efforts were made to reduce bias, where possible, given that data were prospectively acquired from multiple centers with a very low level of missing data. Thus, we were able to include participants with verified diagnoses of depression and/or anxiety, and we were able to adjust the analyses for several potentially confounding covariates at the patient and procedure levels, including baseline alcohol, opioid, and cannabinoid use. Furthermore, we feel our results are generalizable, given that participants were enrolled at a mix of nine community and academic centres across Canada, the United States, and Europe.

## Conclusions

In conclusion, pre-existing depression and/or anxiety clearly negatively impact the patient-reported tolerability of ERCP performed under conscious sedation. More research is needed to further explore the differences in peri-procedural tolerability of ERCP for patients with depression/anxiety and investigate strategies to both enhance tolerability and prevent unplanned healthcare usage following the procedure. Further research is also crucial to assess whether the reduced tolerability reported by patients with mental health disorders during ERCP has negative impacts on patient satisfaction, willingness to undergo future procedures, and direct and indirect healthcare costs.

## Supplementary Information

Below is the link to the electronic supplementary material.

Supplementary Material 1

## Data Availability

The datasets used and/or analysed during the current study are available from the corresponding author on reasonable request.

## Abbreviations

AE: Adverse event
AOR: Adjusted odds ratio
CI: Confidence interval
ERCP: Endoscopic retrograde cholangiopancreatography
PREM: Patient-reported experience measure
PROM: Patient-reported outcome measure
PRO-STEP: Patient-Reported Scale for Tolerability of Endoscopic Procedures
PTSD: Post-traumatic stress disorder
