# Supplementary material for: Patient-reported tolerability of endoscopic retrograde cholangiopancreatography using conscious sedation in patients with underlying depression and anxiety
Source: J Patient Rep Outcomes. 2026 Jun 8;10:143. doi: 10.1186/s41687-026-01094-1 (PMC13473044; doi:10.1186/s41687-026-01094-1)
Supplement: Supplementary file 1 [file 41687_2026_1094_MOESM1_ESM.docx]

**Supplementary Table 1. Unadjusted and adjusted odds ratios between underlying depression and PRO-STEP scores of >3 and >6.**

|  | **PRO-STEP >3^c^** | | | **PRO-STEP >6^c^** | | |
| --- | --- | --- | --- | --- | --- | --- |
| **Variables** | **Unadjusted OR**  **(95% CI)** | **Adjusted OR^a^**  **(95% CI)** | **p value^b^** | **Unadjusted OR**  **(95% CI)** | **Adjusted OR^a^**  **(95% CI)** | **p value^b^** |
| **Intra-procedural domains** | | | | | | |
| Awareness | 1.40 (1.08, 1.82) | 1.22 (0.91, 1.64) | 0.191 | 1.68 (1.23, 2.29) | 1.62 (1.18, 2.22) | **0.003** |
| Discomfort Level | 1.36 (1.01, 1.83) | 1.21 (0.89, 1.66) | 0.226 | 1.81 (1.22, 2.66) | 1.60 (1.06, 2.39) | **0.024** |
| **Post-procedural domains** | | | | | | |
| Abdominal Pain | 1.62 (1.18, 2.22) | 1.44 (1.01, 2.05) | **0.045** | 1.54 (0.97, 2.45) | 1.34 (0.82, 2.20) | 0.241 |
| Throat Pain | 0.95 (0.55, 1.64) | 0.80 (0.46, 1.39) | 0.433 | 0.99 (0.35, 2.78) | 0.77 (0.27, 2.16) | 0.614 |
| Nausea | 2.40 (1.62, 3.54) | 1.97 (1.32, 2.95) | **0.001** | 1.60 (0.86, 2.98) | 1.26 (0.67, 2.36) | 0.469 |
| Distention Level | 1.52 (0.90, 2.56) | 1.90 (1.06, 3.38) | **0.031** | 1.83 (0.70, 4.77) | 2.75 (0.99, 7.66) | 0.052 |

Abbreviations: ERCP, endoscopic retrograde cholangiopancreatography; PRO-STEP, Patient-Reported Scale for Tolerability of Endoscopic Procedures; OR, odds ratio; CI, confidence intervals.

^a^Adjusted for age and sex, and potentially one or more of these covariates: disposition, Charlson comorbidity index, indication, opioids, cannabis, alcohol consumption, previous ERCP, sphincterotomy, targeted duct, size of the CBD, pancreatic cannulation, pancreatogram, stricture present on ERCP, metal stent deployed, difficult cannulation.

^b^p-value associated with adjusted OR in logistic regression model

^c^Binary variable (e.g., yes or no) in which the reference group corresponds to the absence of that characteristic

**Supplementary Table 2. Unadjusted and adjusted odds ratios between underlying anxiety and PRO-STEP scores of >3 and >6.**

|  | **PRO-STEP >3^c^** | | | **PRO-STEP >6^c^** | | |
| --- | --- | --- | --- | --- | --- | --- |
| **Variables** | **Unadjusted OR**  **(95% CI)** | **Adjusted OR^a^**  **(95% CI)** | **P value^b^** | **Unadjusted OR**  **(95% CI)** | **Adjusted OR^a^**  **(95% CI)** | **p value^b^** |
| **Intra-procedural domains** | | | | | | |
| Awareness | 1.78 (1.36, 2.34) | 2.00 (1.50, 2.68) | **0.001** | 1.72 (1.23, 2.39) | 1.76 (1.23, 2.51) | **0.002** |
| Discomfort Level | 1.47 (1.07, 2.01) | 1.31 (0.94, 1.83) | 0.106 | 2.13 (1.43, 3.16) | 1.96 (1.31, 2.94) | **0.001** |
| **Post-procedural domains** | | | | | | |
| Abdominal Pain | 1.79 (1.28, 2.48) | 1.59 (1.06, 2.39) | **0.026** | 1.55 (0.95, 2.54) | 1.08 (0.63, 1.84) | 0.787 |
| Throat Pain | 1.44 (0.87, 2.38) | 1.27 (0.74, 2.21) | 0.387 | 1.90 (0.80, 4.52) | 2.07 (0.83, 5.20) | 0.121 |
| Nausea | 2.47 (1.64, 3.73) | 2.19 (1.38, 3.47) | **0.001** | 2.76 (1.58, 4.80) | 3.37 (1.73, 6.56) | **0.001** |
| Distention Level | 1.84 (0.37, 4.04) | 2.10 (0.16, 28.30) | 0.576 | 1.22 (0.37, 4.04) | 2.07 (0.58, 7.36) | 0.261 |

Abbreviations: ERCP, endoscopic retrograde cholangiopancreatography; PRO-STEP, Patient-Reported Scale for Tolerability of Endoscopic Procedures; OR, odds ratio; CI, confidence intervals.

^a^Adjusted for age and sex, and potentially one or more of these covariates: disposition, Charlson comorbidity index, indication, opioids, cannabis, alcohol consumption, previous ERCP, sphincterotomy, targeted duct, size of the CBD, pancreatic cannulation, pancreatogram, stricture present on ERCP, metal stent deployed, difficult cannulation.

^b^p-value associated with adjusted OR in logistic regression model

^c^Binary variable (e.g., yes or no) in which the reference group corresponds to the absence of that characteristic

**Supplementary Table 3. Unadjusted and adjusted odds ratios between depression and/or anxiety and PRO-STEP scores of >3 and >6 among outpatients only.**

|  | **PRO-STEP >3** | | | **PRO-STEP >6** | | |
| --- | --- | --- | --- | --- | --- | --- |
| **Variables** | **Unadjusted OR**  **(95% CI)** | **Adjusted OR**  **(95% CI)** | **p value** | **Unadjusted OR**  **(95% CI)** | **Adjusted OR**  **(95% CI)** | **p value** |
| **Intra-procedural domains** | | | | | | |
| Awareness | 1.27 (0.93, 1.73) | 1.30 (0.95, 1.79) | 0.104 | 1.26 (0.85, 1.86) | 1.12 (0.71,1.77) | 0.631 |
| Discomfort Level | 1.01 (0.70, 1.48) | 0.77 (0.27, 2.18) | 0.623 | 1.34 (0.81, 2.23) | 1.21 (0.72, 2.03) | 0.460 |
| **Post-procedural domains** | | | | | | |
| Abdominal Pain | 1.61 (1.14, 2.28) | 1.36 (0.94, 1.98) | 0.104 | 1.22 (0.71, 2.10) | 0.96 (0.55, 1.68) | 0.895 |
| Throat Pain | 1.01 (0.57, 1.80) | 0.85 (0.47, 1.52) | 0.581 | 1.01 (0.35, 2.91) | 1.25 (0.41, 3.80) | 0.696 |
| Nausea | 2.65 (1.73, 4.05) | 2.20 (1.43, 3.40) | **0.001** | 2.37 (1.31, 4.31) | 1.82 (0.99, 3.34) | 0.053 |
| Distention Level | 1.74 (0.94, 3.24) | 1.94 (0.95, 3.92) | 0.067 | 1.01 (0.23, 4.48) | 1.19 (0.25, 5.56) | 0.825 |

Abbreviations: ERCP, endoscopic retrograde cholangiopancreatography; PRO-STEP, Patient-Reported Scale for Tolerability of Endoscopic Procedures; OR, odds ratio; CI, confidence intervals.

^a^Adjusted for age and sex, and potentially one or more of these covariates: Charlson comorbidity index, indication, opioids, cannabis, alcohol consumption, previous ERCP, sphincterotomy, targeted duct, size of the CBD, pancreatic cannulation, pancreatogram, stricture present on ERCP, metal stent deployed, difficult cannulation.

^b^p-value associated with adjusted OR in logistic regression model

^c^Binary variable (e.g., yes or no) in which the reference group corresponds to the absence of that characteristic
